# Dramatic Increases in Telehealth-Related Tweets during the Early COVID-19 Pandemic: A Sentiment Analysis

**DOI:** 10.3390/healthcare9060634

**Published:** 2021-05-27

**Authors:** Tiffany Champagne-Langabeer, Michael W. Swank, Shruthi Manas, Yuqi Si, Kirk Roberts

**Affiliations:** School of Biomedical Informatics, The University of Texas Health Science Center, 7000 Fannin Suite 600, Houston, TX 77030, USA; Michael.W.Swank@uth.tmc.edu (M.W.S.); shruthi.manas@uth.tmc.edu (S.M.); yuqi.si@uth.tmc.edu (Y.S.); Kirk.Roberts@uth.tmc.edu (K.R.)

**Keywords:** telehealth, telemedicine, Twitter, NLP, COVID-19

## Abstract

The COVID-19 pandemic resulted in a large expansion of telehealth, but little is known about user sentiment. Tweets containing the terms “telehealth” and “telemedicine” were extracted (n = 192,430) from the official Twitter API between November 2019 and April 2020. A random subset of 2000 tweets was annotated by trained readers to classify tweets according to their content, including telehealth, sentiment, user type, and relation to COVID-19. A state-of-the-art NLP model (Bidirectional Encoder Representations from Transformers, *BERT*) was used to categorize the remaining tweets. Following a low and fairly stable level of activity, telehealth tweets rose dramatically beginning the first week of March 2020. The sentiment was overwhelmingly positive or neutral, with only a small percentage of negative tweets. Users included patients, clinicians, vendors (entities that promote the use of telehealth technology or services), and others, which represented the largest category. No significant differences were seen in sentiment across user groups. The COVID-19 pandemic produced a large increase in user tweets related to telehealth and COVID-19, and user sentiment suggests that most people feel positive or neutral about telehealth

## 1. Introduction

The novel Coronavirus (COVID-19) was first identified in Wuhan, China, and rapidly spread across the globe, creating a public health crisis of unprecedented proportions [1]. The COVID-19 pandemic has catalyzed dramatic increases in telehealth utilization, and in the United States (US), many regulatory constraints have been relaxed to facilitate safer, contact-free access to healthcare [2]. Telehealth capabilities are vast, ranging from simple telephone consultations while consulting a patient’s medical documentation to more complex live-video conferencing, remote monitoring, and diagnostic assessment. In this study, we use the broadest definition of telehealth to include telemedicine. Although several effective vaccines are now in distribution and herd immunity is a current target, some measure of social distancing will remain in effect in 2021. Telehealth will thus continue to be an effective tool for the delivery of healthcare while reducing the risk of infection that may come from in-person contact between patient and provider [3].

During the current global pandemic, people have become more active on social media as an outlet for communication, and mobile technology has increasingly become more important as a tool for government and health organizations to disseminate information [4,5]. Twitter is the most popular microblogging platform in the US and provides a rich source of data for determining the insights and thoughts of its users. Twitter was used successfully by researchers to detect emerging public health issues [6]. An important tool in the analysis of social media is Natural Language Processing (NLP), which is broadly defined as the use of computer algorithms to analyze large amounts of human language (predominantly text data) in order to extract meaning [7]. NLP is widely used to examine unstructured data and to determine how patterns manifest through the evaluation of language and key words, especially in social science research [8]. Although NLP has great potential for monitoring public discourse, the reliability and veracity of user-generated tweets remain a concern, as they cannot be validated as either factual or scientific [9]. However, leveraging a methodology from the field of artificial intelligence developed by a team from Google and presently considered the state-of-the-art model in NLP pre-training and language representation, Bidirectional Encoder Representations from Transformers (BERT) allows a significant number of posts to be analyzed in a short period of time with high accuracy [10,11]. As a barometer of public sentiment, Twitter is an ideal platform for analysis, as the focus is not on factual information, but rather on how people feel about trending topics or events. Social networks such as Twitter have the capability to amass a significant number of posts from users across the world, thus generating polarizing opinions, dangerous rumors, or real-time notifications about disaster threats or responses. At its most extreme, this source of data may prove a benefit and possible predictor of cyber-attacks as users express their thoughts and feelings in a public forum [12]. Sentiment, being an expression of an emotional state, can be classified as positive, negative, or neutral. Further analyzing the lexicon within specific tweets can uncover motivational factors for behavior [13]. The global proliferation of social media platforms such as Twitter allows for an endless stream of people to express their opinions and feelings about emerging public health events and has created new opportunities for informatics research to probe the COVID-19 pandemic era zeitgeist [14]. The purpose of this paper is to analyze a large body of tweets both pre-COVID-19 and early-COVID-19 to determine both interest and sentiment regarding telehealth and the influence of COVID-19.

## 2. Materials and Methods

### 2.1. Raw Data

Tweets published between 1 November 2019 and 9 April 2020 containing the terms “telehealth” and “telemedicine” were extracted using the official Twitter application programming interface (API). Our data preparation and analysis workflow consisted of the following steps: (1) Data collection; (2) Exclusions and de-duplications; (3) Preprocessing/qualitative review; (4) Unsupervised machine learning. There were a total of 117,242 telehealth and 88,321 telemedicine tweets collected. After excluding retweets and duplications (i.e., 13,133 tweets contained both terms), the total was further analyzed (n = 192,430). Preprocessing included a qualitative review of a random subset of 2000 tweets for categorical analyses. 

### 2.2. Manual Data Annotation

Prior to NLP-based classification, the raw data had to be “labeled” for supervised machine learning. In total, 2000 tweets were manually double-annotated by a group of three individuals for the following: (1) The relation to telehealth (yes, no); (2) The sentiment (positive, neutral, negative); (3) The user category (clinician, consumer, policymaker, vendor, other); (4) The relationship to COVID-19 (yes, no). A tweet received a positive sentiment if it contained optimistic, encouraging, or validating language, e.g., “Telehealth is a valuable tool to provide care; protect people in this COVID19 pandemic”. A tweet received a negative sentiment code if the tweet contained emotional words that conveyed pessimistic, debasing, or discouraging feelings, e.g. “Do you want people to keep dying and you aren’t doing anything about it?” Finally, a tweet received a neutral sentiment if it included neither negative nor positive words; these tweets frequently expressed educational or objective informational phrases, e.g., “Effects of a telehealth educational intervention on medication adherence”. When annotating telehealth-related tweets for sentiment, there was a possibility that a tweet could mention both telehealth and a sentiment—but have a sentiment unrelated to telehealth. Annotators were trained to evaluate the sentiment only as it related to telehealth, and these data were used to train the machine learning model. 

A user was regarded as a clinician if the tweet contained key phrases which alerted to clinical events or activities, such as “Excited to speak to residents about ethics and telemedicine in medical careers”. A user was regarded as a consumer if the tweet contained phrases signaling they had used the technology as a patient or an obvious third party, such as “Telemedicine is being offered. Have a video session next week.” A user was regarded as a vendor if the tweet included phrases which suggested the user had an economic stake in promoting a product or service, such as “Dermatology Telemedicine Physician seeking Dermatologists to join”. The user was considered a policymaker if a policy, governmental entity, or institutional course of action was discussed in the tweet. The user was considered “other” if the tweet was unable to be easily placed into any one category. Any case-insensitive use of the terms “covid”, “covid-19”, or “coronavirus” indicated a relationship to COVID-19.

The first 200 tweets were manually annotated by all three annotators, then reconciled as a group that included an expert in NLP (KR) to calibrate the annotations. The remaining 1800 tweets were double-annotated by two of the three annotators. Afterward, all disagreements regarding the classification of the tweets were further reconciled to ensure a consistent set of manual annotations. The annotator agreement with the reconciled standard was 0.78 for telehealth (Cohen’s Kappa), 0.78 for COVID-19-related (Cohen’s Kappa), 0.77 for user (Fleiss’s Kappa), and 0.67 for sentiment (Fleiss’s Kappa). All 2000 manually annotated tweets were used to train/evaluate the NLP model, as described in the next section.

### 2.3. Automatic NLP-Based Annotation

In order to categorize the remaining tweets, two NLP models were evaluated. Both were based on BERT, considered the most innovative model in NLP pre-training and language representation [10,11]. The first model was the standard BERT-base model without any domain-specific pre-training. The second model was BERT-base pre-trained (an unsupervised process) on the raw text of the 192,430 tweets (referred to below as BERT-telehealth). These two BERT models were fine-tuned (i.e., supervised training) on all four tasks (telehealth, sentiment, user, COVID-19) using the standard BERT TensorFlow code, resulting in a total of eight fine-tuned models. The models were evaluated by splitting the dataset in the ratio 80:20 (i.e., 4:1 train/test split); thus, 80% of the subset (1600 tweets) was used for training, and 20% of the subset (400 tweets) was used for testing. The purpose of evaluating both BERT-base and BERT-telehealth was to assess whether the additional unsupervised pre-training on this dataset improved the ability of the supervised model to make correct predictions on the four tasks.

Finally, the best-performing model for each of the four tasks (which all happened to be BERT-telehealth models, as described in the Results) was run across the full dataset of 192,430 tweets in order to analyze the overall reaction, on Twitter, to the impact of COVID-19 on telehealth.

## 3. Results

**Figure 1:**
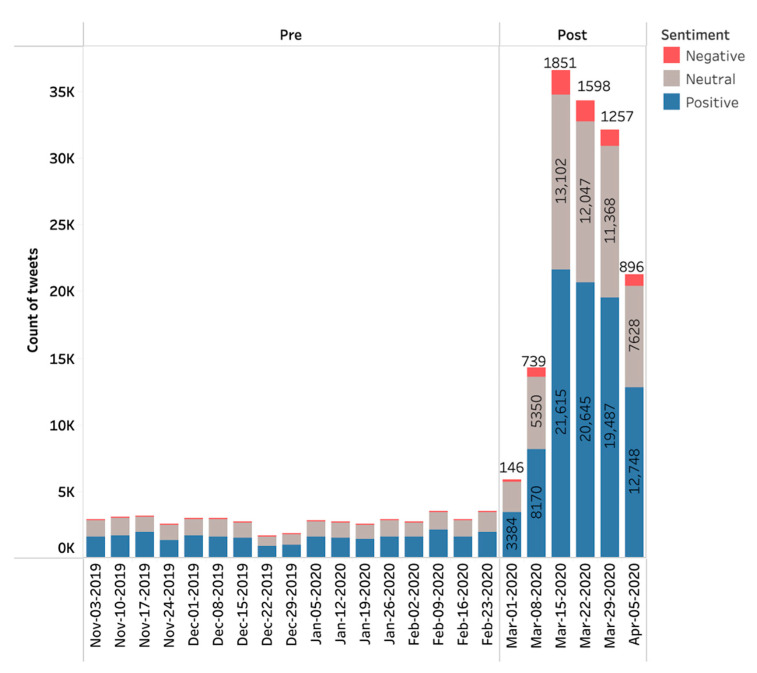
Total weekly tweets and sentiment from November 2019 to April 2020.

From a sample of tweets obtained between November 2019 and April 2020, there was a total of 192,430 tweets related to telehealth and telemedicine. Prior to the COVID-19 outbreak in November 2019, there was a relatively small and stable baseline of telehealth or telemedicine-related tweets. In the initial 4-month period sampled here, there were on average approximately 2700 telehealth-related tweets per week, with slight decreases to 2000 per week in activity during the Thanksgiving and Christmas holidays in 2019. This was followed by a rapid escalation of activity, reaching a peak of 35,625 tweets during the week of 15 March 2020—a nearly 13-fold increase (Figure 1). Given the stable, low baseline activity during the first 4 months, we defined a cutoff point as the week in which total tweets were greater than 1.5 times during the previous 4-week average, which was the week of 1 March 2020; therefore, as shown in Figure 1, *pre* is 3 November 2019 through 28 February 2020, and *post* is from 1 March through 5 April 2020. Throughout the entire period, there were no significant differences in sentiment of positive to neutral to negative from week to week. 

The NLP results for the eight models (two BERT models; four categories) are shown in Table 1. More details, including per-class performance and confusion matrices, can be found in the supplemental material (Tables S1 and S2). For all four categories, the BERT model with additional telehealth-related tweets (BERT-telehealth) outperforms the open-domain BERT model (BERT-base) by a small margin. As a result, all further analyses below were conducted using the output of the BERT-telehealth model on the corpus of 192k tweets. 

As can also be seen from Table 1, while BERT-telehealth is very accurate at assessing whether a tweet with the telehealth keywords is in fact relevant to telehealth (98.5%), and is still quite accurate at assessing whether a tweet is related to COVID-19 (94.9%), accuracy is lower on both sentiment (70.4%) and user (69.0). Part of the reason for the lower accuracies is that, unlike telehealth and COVID-19 classifications, these are not binary classifications (sentiment has three classes, user has five) which makes the task more challenging. The larger issue, however, is the ambiguity in these tweets for these categories due to the short length of the text, which is an inherent characteristic of tweets. Since the BERT-telehealth model outperforms BERT-base in almost every case, we used this model for all subsequent analyses.

Table 2 shows the distribution of tweets by user type, with three-fourths of the coded tweets (75.1%) classified as “other”, followed by 12.9% tweets as “vendor”. Clinicians represented 7.9%, tweets from consumers were 3.3%, and tweets from policymakers represented the lowest category of the users with just 0.8% of tweets (Table 2).

Table 3 shows the distribution of all user tweets by sentiment. Overall, the majority of the tweets presented either a positive (58.6%) or neutral (37.6%) sentiment, with a small remaining portion (3.8%) conveying a negative sentiment (Table 3). Tweets were redacted to protect user anonymity. 

**Figure 2:**
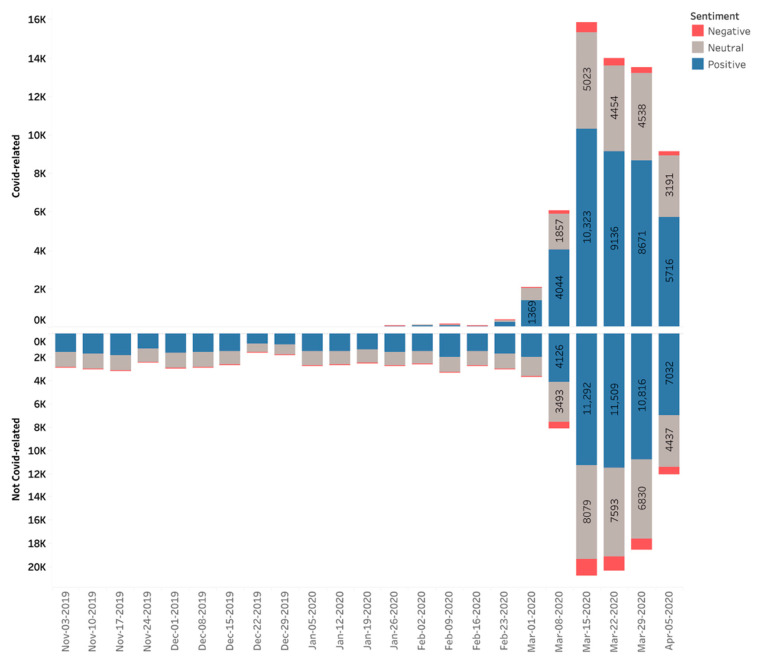
Telehealth tweets related to COVID-19 and unrelated to COVID-19.

Prior to the pandemic entering Europe or the United States regions, few of the telehealth or telemedicine-related tweets referred to COVID-19 (Figure 2). Once COVID-19 became more salient due to increased awareness, COVID-19 related tweets began to increase dramatically, especially after 1 March 2020. Even as overall telehealth and telemedicine-related tweets increased, the division between COVID-19 and non-COVID-19 related tweets was roughly symmetrical, with no obvious differences in sentiment, which remained overwhelmingly positive or neutral for both categories. 

**Figure 3:**
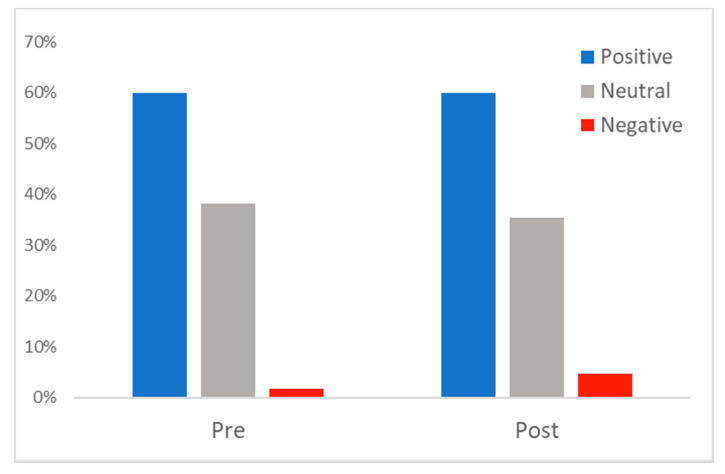
Consumer sentiment of tweets analyzed pre- and post- 1 March 2020.

Given that consumers as the end-users are the ones most likely to benefit from telehealth and telemedicine, we examined user sentiment from consumers only. Although overall sentiment in telehealth-related tweets across all users was overwhelmingly positive, it would seem unlikely that, for example, vendors would express a negative sentiment on a social media platform [15]. As Figure 3 shows, consumers showed mostly positive (60.0%) or neutral (38.2%) sentiment before 1 March 2020 (pre-), and this was essentially the same post-, with 59.9% positive and 35.5% neutral. There was a slight shift from neutral to negative, with negative sentiment in telehealth-related tweets among consumers increasing from 1.8% pre- to 4.6% post-. 

**Figure 4:**
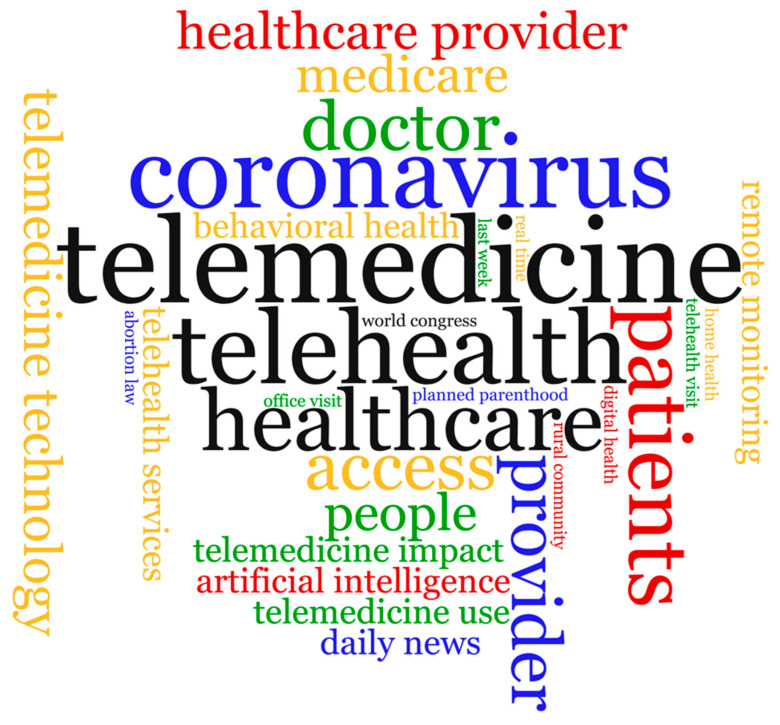
Word cloud visualization of most used telehealth-related bigrams and unigrams found in tweets between November 2019 and April 2020.

We further analyzed the text of the 192,430 tweets for the most used bigrams and unigrams (single words) and displayed them in a word cloud visualization (Figure 4). Bigrams are pairs of adjacent words that may or may not be grammatically correct or have semantic meaning [16]. The top five unigrams by frequency from 1 to 5 were telemedicine, telehealth, coronavirus, healthcare, and patients; while the top five bigrams were telemedicine technology, telehealth services, behavioral health, healthcare provider, and remote monitoring. 

## 4. Discussion

In this study, we analyzed 192,430 publicly posted messages related to telehealth and telemedicine during a time of heightened awareness and demand for this healthcare platform. The first finding from this work is that telehealth tweets were low and stable during the 4 months (November 2019–February 2020) preceding the announcement of social distancing guidelines, followed by a dramatic increase in activity during the week of 1 March 2020. This increase correlated with news coverage of the pandemic, and in particular the announcement from the World Health Organization that COVID-19 was determined to be a pandemic [17]. 

Although telehealth and telemedicine were utilized for a number of years prior to the COVID-19 pandemic, interest dramatically increased as evidenced by telehealth-related tweets shortly after the onset of the pandemic. Surveys of individuals conducted during this same period also showed favorable views by healthcare providers; however, there were concerns expressed regarding patients with public versus private insurance [18]. Although it is likely that COVID-19 was the impetus for increased telehealth tweet activity, this generated two different types of interest in telehealth. The first was that people were tweeting about telehealth and COVID-19 because they were concerned about the pandemic, seeking testing, wanting to see their doctor, considering they might have COVID-19, or other information-seeking requests related to COVID-19. The other reason people may have used Twitter stemmed from the need for social distancing, implemented either voluntarily or through specific policies—including the healthcare delivery system and access to physicians, regardless of the symptoms or disease. These tweets were not directly related to COVID-19, but rather indirectly related due to the pandemic’s impact on the wider healthcare system. 

The next finding was that sentiment remained mostly positive, followed by neutral, with low negative ratings. The distributions of sentiment did not change significantly despite a substantial increase in volume. Thus, the pandemic increased overall interest in telehealth without changing sentiment. This has implications for those in the field of telemedicine, as patients may share openly about their experiences using social media in an unstructured way, as opposed to using structured surveys [19]. Further, a vendor seeking to gain traction in a new market or policymaker looking to draw attention to new initiatives could follow a similar approach to other social media outlets, aligning themselves with reputable, influential users with large numbers of followers or a significant volume of tweets [20]. 

There are limitations to using NLP as opposed to manual annotations. While using a machine-learning model is a feasible way to categorize a large number of tweets and allows for a democratization of language, there are limits to the veracity of classification. Notably, as shown in Table 1, the NLP results are far from perfect, which could effectively introduce a sample bias into the downstream analysis. The largest user category defined here is *Other*, with 75% of total tweets, which suggests that there was not enough information in the tweets to make a definitive user categorization without defaulting to more identifiable meta-data. In February–March 2020, as healthcare became more critical and less accessible during the height of the pandemic, it is possible more users who were less familiar with the platform were driven to Twitter to seek information. The inability to strategically place users into groups may suggest the classification algorithm is unable to identify relevant new users, thus increasing the classification category of “other” by the NLP pre-training model. Studies have concurred that healthcare has proven to be a challenging field for social media and sentiment analysis due to the high usage of nouns, low self-identification, and more objective speech [21,22]. These results were also seen in our bigram and unigram analysis. This means the model did not always make an accurate prediction of user type, despite the relative precision at which it was able to categorize the topic of the tweet. For example, many users may post information about a particular product that they like; however, the model may categorize these users as vendors, when in fact they are consumers discussing a product. Although consumers represented one of the smallest categories in terms of total tweets, they frequently used phrases such as “my doctor” or “my appointment” which allowed the model to make a more accurate classification as user. Lastly, it is possible that a user posted a tweet about telehealth then express an unrelated sentiment. Although the annotators were trained to catch this abnormality, the NLP model may have missed this. Therefore, there is a possibility that some of the telehealth-related tweets in this study had sentiments unrelated to telehealth.

Other research has assessed the sentiment specifically from healthcare providers and showed similar results. Tweets from providers were mostly positive but had themes specific to access to telemedicine and the safety of telemedicine as a delivery mechanism [23]. Future research could attempt to qualitatively analyze the tweets according to themes, thus providing additional data on the topics discussed. This additional category might allow for inter-category relationships to be established, such as consumers seeking specific information regarding telemedicine.

## 5. Conclusions

Social media platforms such as Twitter have provided a conduit for public sentiment during the COVID-19 pandemic. In this research, we analyzed a large body of tweets related to telemedicine and telehealth during the initial quarantine period before and after March 2020. Using unsupervised NLP methodologies, we were able to show that sentiment towards this delivery modality remained positive or neutral despite a significant increase in volume.

## Figures and Tables

**Table 1:** Evaluation of the eight fine-tuned BERT models on the test set. Note: sentiment and user are not binary, so precision/recall/F1 are macro metrics and AUROC is not applicable.

	BERT Model	Accuracy	Precision	Recall	F1	AUROC *
Telehealth	*BERT-base*	98.3%	98.5%	99.7%	99.1	0.982
	*BERT-telehealth*	98.5%	98.8%	99.5%	99.2	0.989
Sentiment	*BERT-base*	67.8%	63.6%	56.3%	58.8	N/A
	*BERT-telehealth*	70.4%	70.0%	61.7%	64.5	N/A
User	*BERT-base*	67.5%	53.8%	53.7%	53.7	N/A
	*BERT-telehealth*	69.0%	57.6%	54.7%	56.0	N/A
COVID-19	*BERT-base*	93.6%	91.3%	83.2%	87.1	0.940
	*BERT-telehealth*	94.9%	94.5%	85.2%	89.6	0.952

* AUROC, area under the receiver operating characteristic: evaluation metric utilized to determine the model’s performance.

**Table 2:** Distribution of tweets by user type.

Category	Definition	User Count	(%)
Clinician	A person who treats patients	15,136	(7.9)
Consumer	A patient or other user of telehealth	6381	(3.3)
Policymaker	A person who makes or influences governmental policy	1544	(0.8)
Vendor	Any user with an economic interest in telehealth	24,888	(12.9)
Other	Any other user who cannot be classified as above	144,481	(75.1)

**Table 3:** Distribution of tweets by sentiment.

Category	Definition	Example Tweet	n	(%)
Positive	Supports use of telehealth	*Telehealth may be especially helpful as an initial option for COVID-19*	112,721	(58.6)
Neutral	No overt positive or negative sentiment	*Telehealth During COVID-19: New Rules and Considerations*	72,369	(37.6)
Negative	Dissatisfaction with telehealth	*I have a telehealth appointment with my tomorrow and it’s going to be so weird*	7340	(3.8)

## Data Availability

Data sharing is not applicable.

## Supplementary Materials

The following are available online at https://www.mdpi.com/article/10.3390/healthcare9060634/s1, Table S1: BERT-base, Table S2: BERT-telehealth.
