# Supplementary material for: Dramatic Increases in Telehealth-Related Tweets during the Early COVID-19 Pandemic: A Sentiment Analysis
Source: Healthcare (Basel). 2021 May 27;9(6):634. doi: 10.3390/healthcare9060634 (PMC8230122; doi:10.3390/healthcare9060634)
Supplement: Supplementary file 1 [file healthcare-09-00634-s001.zip › healthcare-1218018-supplementary.pdf]

Table S1: BERT-base

COVID

	Predicted positive	Predicted negative
Actual positive	TP=84	FN=17
Actual negative	FP=8	TN=279

Telehealth

	Predicted positive	Predicted negative
Actual positive	TP=391	FN=1
Actual negative	FP=6	TN=2

User

		**Predicted**				
		**Unknown**	**Vendor**	**Clinician**	**Consumer**	**Policymaker**
**Actual**	**Unknown**	210	41	9	4	2
	**Vendor**	43	29	2	1	0
	**Clinician**	7	4	9	4	0
	**Consumer**	7	0	0	12	0
	**Policymaker**	2	0	0	0	2

	Recall	Precision	F1
**Unknown**	0.7895	0.7807	0.7850
**Vendor**	0.3867	0.3919	0.3893

**Clinician**	0.3750	0.4500	0.4091
**Consumer**	0.6316	0.5714	0.6000
**Policymaker**	0.5000	0.5000	0.5000

	Train	Test	Total
**Unknown**	1091	266	1357
**Vendor**	279	75	354
**Clinician**	93	24	117
**Consumer**	71	19	90
**Policymaker**	19	4	23

Sentiment

		**Predicted**		
		**4**	**3**	**2**
**Actual**	**4**	178	39	4
	**3**	65	77	2
	**2**	9	6	8

	Recall	Precision	F1
**4**	0.8054	0.7063	0.7526
**3**	0.5347	0.6311	0.5789
**2**	0.3478	0.5714	0.4324

	Train	Test	Total
**4**	886	221	1107
**3**	577	144	721
**2**	90	23	113

Table S2: BERT-telehealth

COVID

	Predicted positive	Predicted negative
Actual positive	TP=86	FN=15
Actual negative	FP=5	TN=282

Telehealth

	Predicted positive	Predicted negative
Actual positive	TP=390	FN=2
Actual negative	FP=4	TN=4

User

		Predicted				
		**Unknown**	**Vendor**	**Clinician**	**Consumer**	**Policymaker**
**Actual**	**Unknown**	212	41	8	3	2
	**Vendor**	38	33	3	1	0
	**Clinician**	10	3	10	1	0
	**Consumer**	8	0	0	11	0
	**Policymaker**	2	0	0	0	2

	Recall	Precision	F1
**Unknown**	0.7970	0.7852	0.7910
**Vendor**	0.4400	0.4286	0.4342
**Clinician**	0.4167	0.4762	0.4444

**Consumer**	0.5789	0.6875	0.6286
**Policymaker**	0.5000	0.5000	0.5000

Sentiment

		**Predicted**		
		**4**	**3**	**2**
**Actual**	**4**	185	31	5
	**3**	67	77	0
	**2**	8	4	11

	Recall	Precision	F1
**4**	0.8371	0.7115	0.7692
**3**	0.5347	0.6875	0.6016
**2**	0.4783	0.6875	0.5641
